# Candidate Binding Sites for Allosteric Inhibition of the SARS-CoV-2 Main Protease from the Analysis of Large-Scale Molecular Dynamics Simulations

**DOI:** 10.1021/acs.jpclett.0c03182

**Published:** 2020-12-11

**Authors:** Matteo Carli, Giulia Sormani, Alex Rodriguez, Alessandro Laio

**Affiliations:** † Scuola Internazionale Superiore di Studi Avanzati (SISSA), Via Bonomea 265, 34136 Trieste, Italy; ‡ The Abdus Salam International Centre for Theoretical Physics (ICTP), Str. Costiera, 11, 34151 Trieste, Italy

## Abstract

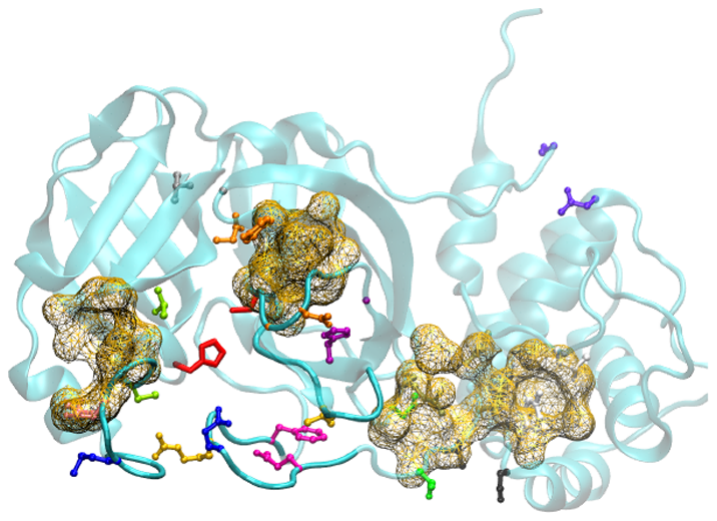

We analyzed a 100 μs MD trajectory of the SARS-CoV-2 main protease by a non-parametric data analysis approach which allows characterizing a free energy landscape as a simultaneous function of hundreds of variables. We identified several conformations that, when visited by the dynamics, are stable for several hundred nanoseconds. We explicitly characterize and describe these metastable states. In some of these configurations, the catalytic dyad is less accessible. Stabilizing them by a suitable binder could lead to an inhibition of the enzymatic activity. In our analysis we keep track of relevant contacts between residues which are selectively broken or formed in the states. Some of these contacts are formed by residues which are far from the catalytic dyad and are accessible to the solvent. Based on this analysis we propose some relevant contact patterns and three possible binding sites which could be targeted to achieve allosteric inhibition.

The severe acute respiratory syndrome which broke out in December 2019 (COVID-19) is caused by coronavirus 2 (SARS-CoV-2).^1,2^ Its main protease (M^pro^ or 3CL^pro^) was the first protein of SARS-CoV-2 to be crystallized, in complex with a covalent inhibitor, in January 2020.^3^ It is essential in the viral life cycle since it operates at least 11 cleavage sites on large viral polyproteins that are required for replication and transcription,^3,4^ so it is an attractive target for the design of antiviral drugs.^5^ Since there is no known human protease having a cleavage specificity similar to the one of M^pro^, it may be possible to design molecules that do not interact with human enzymes.^3,4^

M^pro^ is a homodimer. Each monomer has 306 residues and is composed of three domains. Domains I and II (residues 10–99 and 100–182, respectively) have an antiparallel β-barrel structure. The binding site of the substrate is enclosed between these β-sheets.^4^ Domain III (residues 198–303) contains five α-helices and has a role in the regulation of the protein dimerization.^4^ The two residues His^41^ and Cys^145^ form the catalytic dyad. The structure and way of functioning of the SARS-CoV-2 M^pro^ are similar to those of the SARS-CoV M^pro^.^6,7^ This is expected, due to a 96% sequence identity between them.

The most direct strategy to block the action of the M^pro^ is through small molecules that directly interact with the catalytic site. The first *in silico* trials were made with covalent inhibitors known to be interacting with the catalytic site of SARS-CoV M^pro^, such as N3^3^ or 11r^4^. Many efforts followed in the field of virtual screening. In this kind of studies, computational docking of millions of molecules is performed, and the behavior of the best candidates is usually then tested through MD simulation.^8−13^ Another possible route that can be followed to stop the action of the M^pro^ is allosteric inhibition.^14,15^ The functional definition of allosteric regulation implies the energetic coupling between two binding events.^16,17^ The binding of the allosteric ligands affects orthosteric pockets by altering protein dynamics, either through large-scale structural changes or through more subtle changes in correlated residue motions.^18,19^ Following the idea of conformational selection,^20^ allosteric effectors will act as inhibitors by stabilizing configurations in which the access to the active pocket is at least partially closed. In short, the idea is to block the protease in one of its metastable conformations, in which the catalytic dyad cannot regularly operate, inhibiting in this way the whole protein functionality. This approach, at least in principle, has several advantages. First of all, it offers the possibility to drug sites far from the catalytic pocket, thus enlarging the chance to discover active compounds and to obtain non-competitive inhibition. If an allosteric site is identified and targeted, using this strategy, one can develop drugs which are highly specific since they do not bind in active sites, which are typically conserved in protein families.^21^ Owing to these advantages, allostery has been established as a mechanism for drug discovery, for example to target G-protein-coupled receptors (GPCRs)^22,23^ or protein kinases.^24−26^

We here propose a strategy to identify candidate binding sites for allosteric inhibition which is fully based on the analysis of a long molecular dynamics (MD) trajectory. We analyze a 100 μs MD trajectory of the M^pro^ generated in the D. E. Shaw Lab.^27^ Our scope is to search for possible metastable states of the protease, namely configurations which do not change significantly on the scale of several tens of ns. These configurations are important for developing drugs for allosteric inhibition, since they are already (marginally) stable, and by designing a ligand which increases their stability, they can become kinetic traps.^21^ These metastable states are searched by an approach, developed by us, which allows estimating the free energy landscape of a system in a high dimensional space.^28,29^ The local minima of the free energy, if deep enough, correspond to the metastable states, approximately the same that would be found by performing a much more expensive Markov State Modeling analysis.^30^

The competitive advantage of our approach is that it allows performing the analysis in very high-dimensional spaces, taking into account at the same time several hundred different variables. This allows finding the free energy minima, and thus the metastable states, with no prejudice on their structure. In the case of the M^pro^, we carry out our analysis in two different spaces: the space defined by all the ψ backbone dihedrals of the protease, and the space defined by the contacts between pairs of residues which break or form during the dynamics. Both spaces consider the enzyme globally, not limiting the analysis to the catalytic dyad or to the binding pocket, which is essential to unveil possible allosteric states. Based on a characterization of global and local properties of these states, we propose a few possible targets which could serve as binding sites for drug-like compounds with the purpose of allosteric inhibition.

We extract from the 100 μs MD trajectory 10 000 equally spaced frames, one every 10 ns. Since the enzyme is a homodimer, we consider the 20 000 total frames of the two monomer trajectories as a sample of the conformational space of a single monomer. However, the trajectories of the two monomers are considered and analyzed separately, in order to verify *a posteriori* whether the configurations they explore are similar or not.

In both metric spaces in which we perform our analysis, we neglect the 10 residues at the C-terminus of the peptide, since they are highly mobile in both monomers and might introduce noise in the analysis. The two metrics are

- the ψ*-backbone-dihedral distance*:^31^ such distance between configurations *t* and *t*′ is defined as θ_*t*,*t*′_ = ∑*_i_*((ψ_*i*,*t*_ – ψ_*i*,*t*′_))^2^, where ψ_*i*,*t*_ is the value at time *t* of the ψ dihedral angle that involves the α-carbon of residue *i* of the monomer, index *i* runs between 1 and 296, and the notation ((·)) stands for 2π-periodicity within the brackets;
- the *contact-map distance*,^31^ restricted only to contacts which vary significantly during the simulation. To define these mobile contacts, we first compute the contact-map matrix *C* for each frame, restricted to residues 1–296. For each couple of residues *ij*, we first evaluate the distances between all the couples of heavy atoms, with one atom belonging to *i* and the second one belonging to *j*. *C*_*ij*_ is then equal to σ(*d*_min_), where *d*_min_ is the smallest distance between the couples of atoms and σ is the sigmoidal function: σ = (1 – (*d*/*r*_0_)^10^)/((1 – (*d*/*r*_0_)^20^)), with *r*_0_ = 4.5 Å. We consider as *mobile* the contacts which are completely formed (*C*_*ij*_ > 0.8) in at least 5% of the frames and completely broken (*C*_*ij*_ < 0.2) in at least 5% of the frames. Moreover, we neglect those contacts which have a value between 0.2 and 0.8 (i.e., close to *r*_0_) in more than 50% of the frames. This procedure selects 155 relevant mobile contacts for the first monomer (m1) and 184 for the second (m2). Most of these contacts are in common, which is reasonable since the two monomers are chemically identical; the union of the two sets has 235 elements. Denoting by  the set of mobile contacts of a monomer, the contact-map distance between configurations *t* and *t*′ is *d*_*t*,*t*′_ = , where *C*(*t*) is the contact matrix of configuration *t*.

Our two metrics are both sensitive to local and global conformational changes in the peptide but capture different details: the ψ coordinates keep track of the changes in the protein backbone, whereas the mobile contacts metrics, instead, also keep track of the side-chain rearrangements while neglecting fluctuations around the completely formed or completely unformed contacts.

The free energy landscape of each dataset is estimated following the procedure introduced in ref (32). First of all, the intrinsic dimension (ID) of the manifold containing the configurations is calculated.^33^ In the spaces of the ψ dihedrals we get an ID of 28 for m1 and of 26 for m2. In the spaces of the mobile contacts, we get an ID of 17 for both monomers. The free energy *F* of each configuration is then calculated using the PA*k* estimator,^28^ which also provides an estimate of the uncertainty of *F*. The core of the approach is the calculation of the radius of the neighborhood in which the free energy can be considered constant within a fixed statistical confidence. Importantly, this algorithm requires the knowledge of the ID of the space in which the data points are lying, but it does not require knowing explicitly which variables define the reduced space. Finally, using Density Peak (DP) clustering^34^ in its unsupervised variant,^32^ we build a topography of the free energy landscape. We first find the free energy minima, and we assign all the frames to one of these minima according to the DP procedure. The set of configurations assigned to a single free energy minimum defines a free energy basin. Then, following ref (32), we find the saddle point between each pair of basins. The *core set* (CS) of a basin is the set of configurations whose free energy is lower than the free energy of the lowest saddle point of the basin.

The described approach requires choosing the metric and a single metaparameter, the statistical confidence *Z* at which a basin is considered meaningful. A basin *s* is considered meaningful if (*F*_*ab*_ – *F*_*a*_) > *Z*(ε_*F_a_*_ + ε_*F*_*ab*__) for all the basins *b* which share a border with *a*. Here, *F*_*a*_ is the free energy minimum of basin *a*, ε_*F_a_*_ is its uncertainty, *F*_*ab*_ is the free energy of the saddle point between basin *a* and *b*, and ε_*F_ab_*_ is its uncertainty. In our analysis *Z* is set to the value *Z* = 1.4, which corresponds to a confidence level of approximately 85%. This means that we expect to have nearly 15% of artificially split free energy basins. We verified that, by varying *Z* around this value, the description does not change significantly—the most populated free energy basins remain approximately unchanged.

In the following analysis we call a state a set of configurations which belong to the core set of the same free energy basins according to both metrics. If, for example, a given basin number found using the dihedral metric is split in two different basins according to the contact metric, in our analysis we will consider two states. As a consequence, our states are structurally uniform according to both metrics. We consider in our analysis only states with a population of at least 8 core state configurations. With this criterion, we identify 11 relevant states in the trajectory of m1 and 7 in the trajectory of m2, for a total of 18 metastable states.

First, we want to make sure that the metastable states detected analyzing the m1 and m2 trajectories separately are the same as if we run the algorithm on the merged 20 000 configurations. We check it in the case of the mobile contacts metric. We find that all the clusters involve only frames from the first monomer or only from the second. There is no relevant cluster that shares structures from both monomers, meaning that in terms of the contact map the configurations of m1 are different from the configurations of m2. Due to their chemical identity, in an ergodic simulation the configurations explored by the two monomers should be nearly identical. Therefore, the first important result of our analysis is that 100 μs of MD simulation is not sufficient to explore ergodically all the configuration space, as recently claimed also by Cocina et al.^35^ This is also visible by looking at Figure 1a: most states are visited only two or three times. Consequently, the mean residence time cannot be meaningfully estimated. We instead compute the maximum residence time, considering it a proxy of the metastability of each state. These times are shown in the upper panel of Figure 1b and range from 0.20 to 16.07 μs.

**Figure 1:**
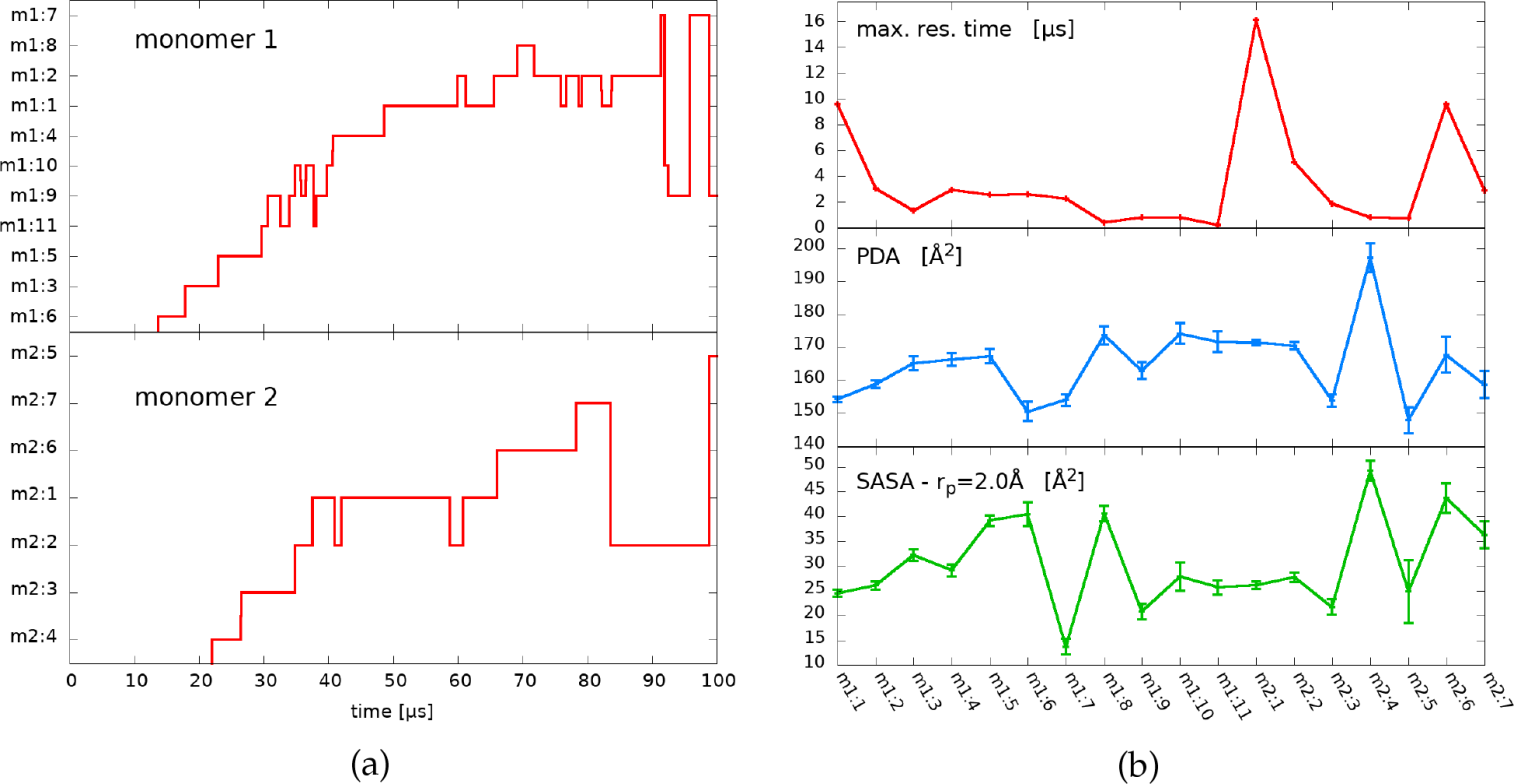
(a) Trajectories for the two monomers in the space of the states. The frames that do not belong to a core set are relabeled by the state identifier of last visited core state; notice there is no label assigned to the first 10–20 μs, indicating that no statistically meaningful metastable state is visited in the first part of the trajectory. (b) Global observables of the states. Top: the maximum residence time for each state, taken as the longest time interval over which the state label does not change. Middle: average PDA of the frames belonging to the core of a state; PDA is defined as the sum of the area of the three triangles formed by the Cα carbons: Thr^25^-Ser^46^-Gly^143^, Ser^46^-Gly^143^-Met^165^, and Gly^143^-Met^165^-Arg^188^, which form the tips of five loops delimiting the cavity (see Supporting Information for a pictorial representation). Bottom: average SASA of the catalytic dyad of the frames belonging to the core of a state; the SASA is computed choosing a probe radius *r*_p_ = 2.0 Å.

To quantify the accessibility to the catalytic site, we estimate the average solvent-accessible surface area (SASA) of the dyad and what we call the *pocket doorway area* (PDA), which quantifies the opening of the catalytic pocket from the position of four selected Cα carbons (see caption of Figure 1b). The two quantities, presented in the middle and lower panels of Figure 1b, are in general quite correlated, although not in all the states. Indeed, contrary to PDA, SASA is sensitive to what happens in the direct proximity of the catalytic residues, while neglecting more macroscopic rearrangements of the catalytic pocket.

Lastly, we characterize the local differences in the states by analyzing in detail their contact structure and their backbone arrangement. In the case of the mobile contacts, we analyze the intramonomer contacts which change significantly between at least 2 of the 18 states; furthermore, we also track the behavior of a few intermonomer contacts that might reflect some changes in the metastable states’ catalytic activity.^6,36^ The contact structure of the selected states is summarized by the table in Figure 2a. As for the backbone, we analyze the ψ dihedral angles in the loops closing the cavity and a few other dihedrals which change significantly in the various states (see Supporting Information).

**Figure 2:**
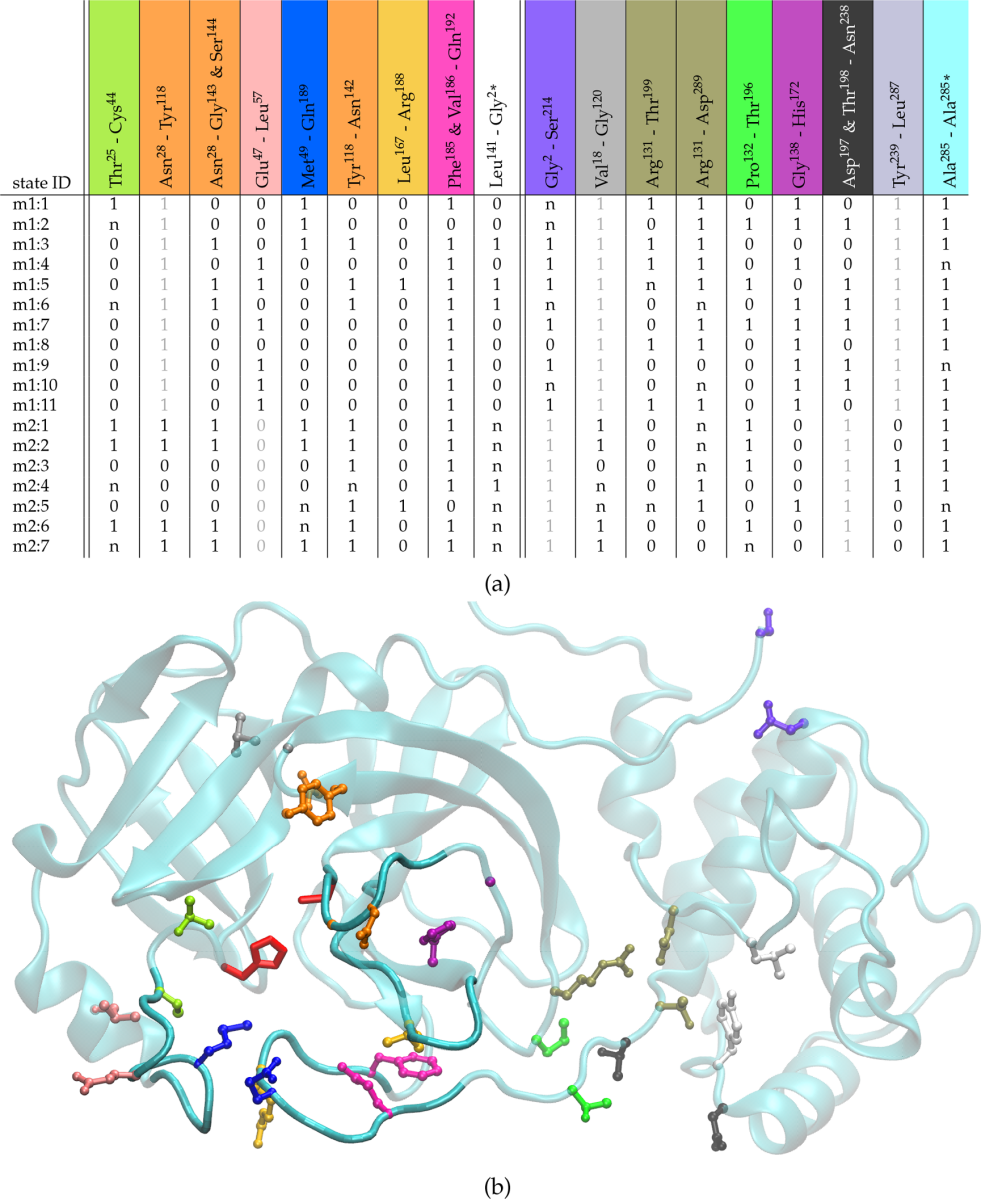
Selected intramonomer contacts and intermonomer contacts (marked with a star (*)). In the case of intermonomer contacts, the residue of the monomer which is excluded by the metric that defines a state is marked with a star (*). For each contact (columns), the average over the configurations of a given state is reported in the corresponding row. Such contacts are divided into two subgroups by a double vertical line: on the left those between residues belonging to the flexible loops which control the access to the binding pocket and on the right the other contacts. For readability, the entries take only three possible labels: 0 when the average over the configurations belonging to a state is <0.3, namely the contact is not formed; 1 when the average is >0.7, namely the contact is formed; *n* in all other case. Contacts whose label does not vary in any of the states of a given monomer are reported in light gray color. The picture shows a VMD^57^ representation of monomeric M^pro^ in state m1:1: on the left-hand side is the enzyme binding pocket, which encloses the catalytic dyad (in red); all other highlighted residue couples refer to the contact with the corresponding color in the table.

As mentioned above, the catalytic dyad His^41^-Cys^145^ is located in the pocket between the protein domains I and II. The access to this cavity is controlled by the flexible loop structures highlighted in Figure 2b. The two most flexible loops^37^ involve residues from Ile^43^ to Pro^52^ (*left flap*) and from Phe^185^ to Tyr^201^ (*linker loop*). The left flap corresponds to the leftmost loop in Figure 2b, and opens and closes like a small door. No conformers from the second dimer m2 have the left flap wide open; consequently, contact Glu^47^-Leu^57^ is never formed. The linker loop closes the cavity from below in Figure 2b and links domains II and III. All the m2 states have a loosely structured linker loop, with contact Arg^131^-Thr^199^ almost never formed and contact Asp^197^ and Thr^198^-Asn^238^ always formed. The contacts controlling the distance between the β barrels of the I and II protein domain^4^ (Asn^28^-Tyr^118^ and Val^18^-Gly^120^), which are always formed in m1, are at times unformed in m2. The loop from Phe^140^ to Cys^145^ (we call it *upper flap*) is smaller and assumes mainly two conformations: tilted downward (contacts Ans^28^-Gly^143^, Ser^144^, and Tyr^118^-Asn^142^ not formed, dihedral ψ_144_ in β configuration), which hides the catalytic Cys^145^, or flat out (ψ_144_ in α configuration), which leaves more access to the dyad. Last, the β-sheet loop from Met^162^ to Gly^170^ delimits the cavity from the right in Figure 2b (we call it *right loop*); it is the least flexible, but it interacts with the N-finger of the other monomer and is crucial for shaping the substrate binding pocket.^38^

All m2 states except m2:5 have the upper flap not tilted down and retracted with respect to the pocket, with contact Tyr^118^-Asn^142^ almost always formed and contact Gly^138^-His^172^ almost never formed. These two contacts are almost always mutually exclusive, with the exception of states m1:6 and m2:5, in which both contacts are formed at the same time. Another important difference among states, not related with the loops, is that dihedrals from Leu^227^ to Asn^238^ (bottom right in Figure 2b) in all states of m1 are arranged in α configuration, so that an α-helix is formed and contact Tyr^239^-Leu^287^ is always formed; in m2 such an α-helix structure is often defective. As for the contact between the N-finger and domain III (contact Gly^2^-Asn^214^), in m2 it is often formed, while it is broken in most m1 states.

We describe all the states in detail in the Supporting Information. Hereby, we focus on the most stable, the most open, and the most closed according to the SASA and PDA observables. From the analysis of the maximum residence time, it is clear that states 1 and 2 of both m1 and m2 are among the longest-lived metastable states. All four are in fact very similar to the crystallographic structure (PDB 6Y84(39)): they all have the left flap and the linker loop in contact between each other (cont. Met^49^-Gln^189^)—the left flap is closed (cont. Glu^47^-Leu^57^ broken, cont. Thr^25^-Cys^44^ formed) and the linker loop stretched toward it (cont. Leu^167^-Arg^188^ broken), covering the lower part of the binding pocket.

The two most open states are m2:4, which ranks the highest in both PDA and SASA, and m1:8. In m2:4 the upper flap is not tilted downward and is far from the pocket and from the right loop, leaving cont. Gly^138^-His^172^ not formed; the left flap is very open (although the dihedrals of this loop are quite variable among the configurations of such state), and the linker loop is slightly contracted (cont. Arg^131^-Thr^199^ and Pro^132^-Thr^196^ not formed), not stretching toward the left flap as in other closed or partly closed states—this leaves the catalytic dyad well exposed. State m1:8 also ranks very high in PDA and in SASA. The left flap is open, although dihedrals from Ile^43^ to Ser^46^ are not all in α configuration; their particular arrangement (αβαc), however, grants that the biggest side chains of the left flap are not oriented toward the binding pocket. The linker loop is not stretched toward the left flap, but rather down, toward the interface with the solvent; it is quite open (dihedral of Gln^189^ in *c* instead of β configuration) in proximity of the pocket, and all its side chains do not obstruct the access to the cavity (in particular those of Arg^188^ and Gln^189^, responsible for a low SASA in other states).

Among the most closed states we mention m1:7, m1:9, m2:3, and m2:5. State m1:9 is very similar to m1:10 in its contact and backbone structure, with the exception of the left flap, which is more open in state m1:10. State m1:9 is also structurally similar to m1:7—the only difference among the contacts is Pro^132^-Thr^196^, which is formed in m1:7 and not in m1:9, allowing the lower loop to be more flexible. In both, the upper flap is tilted downward, but the left flap backbone is open. In m1:9 the side chains of the residues in the loops surrounding the binding pocket are oriented toward the catalytic dyad, causing such state to rank among the lowest in SASA. State m1:7 ranks among the lowest in PDA and as the lowest in SASA; the reason lies in the side chains of the lower and left flaps, in particular of Thr^45^ and Gln^189^, which form a contact and effectively close the access to the reactive site. State m2:3 ranks as the third lowest in both SASA and PDA. Cys^145^ is not well covered, but on the other hand His^41^ is less accessible than in most other states. As most m2 states, m2:3 has the upper flap bent upward and contact Gly^138^-His^172^ not formed. The linker loop is not stretched, leaving the contacts with Arg^131^ partly unformed. The left flap is closed and stretched toward the linker loop, and its dihedrals are arranged in such a way that cont. Met^49^-Gln^189^ is not formed. Finally, state m2:5 is the one with the lowest PDA and is among the lowest-ranked in SASA. Its conformation is quite peculiar: the linker loop is all retracted and coiled (it is the only state of m2 forming cont. Leu^167^-Arg^188^). The left flap is all stretched toward the linker loop (cont. Met^49^-Gln^189^ formed) and almost completely covers the catalytic His^41^. The upper flap, rather than being flat or tilted down, is oriented upward, causing a deformation in domain II which allows cont. Gly^138^-His^172^ to be formed. Remarkably, like m1:9, state m2:5 is one of the few states with cont. Ala^285^-Ala^285*^ not tightly formed.

Our analysis shows that the accessibility to the catalytic dyad is reflected in the forming and breaking of few relevant contacts around the reactive cavity. For example, cont. Glu^47^-Leu^57^ is not formed when the left flap is closed, a condition common to most states in which the catalytic dyad is not accessible. Similarly, the catalytic site (in particular Cys^145^) is less exposed when the upper flap it tilted downward, i.e., when cont. Tyr^118^-Asn^142^ is not formed. The druggability analysis software PockDrug^40^ finds one pocket in correspondence of the residues of each of the two contacts (respectively called left pocket and upper pocket) and assigns to them a druggability probability of 0.68 ± 0.08 and 0.95 ± 0.03. Targeting these two regions with drug-like compounds, blocking the formation of the mentioned contacts, might prove a successful strategy for the inhibition of the catalytic activity. The distribution of SASA over all configurations in which contact Tyr^118^-Asn^142^ is not formed is significantly shifted toward lower SASA values than in the cases in which the contact is formed (see Figure 3b).

**Figure 3:**
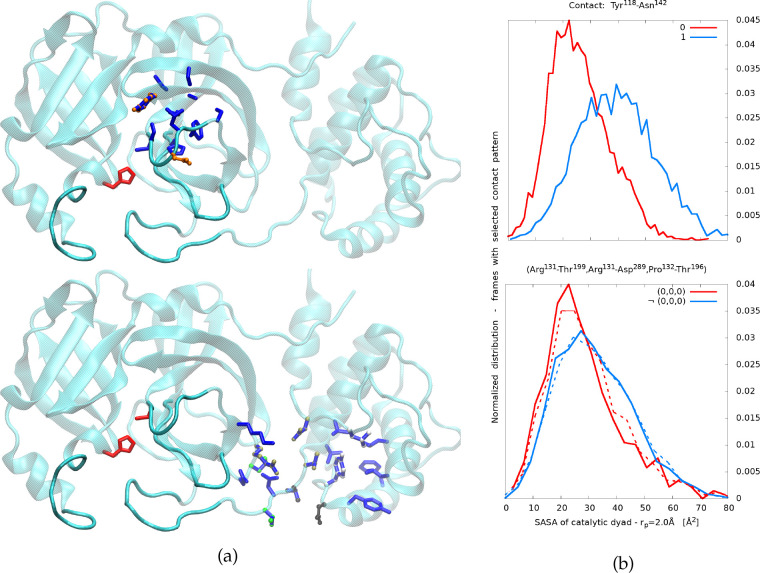
(a) VMD^57^ representation of monomeric M^pro^ in state m1:9: in red is the catalytic dyad, and in dark blue the residues involved in the upper pocket (top) and the distal pocket (bottom) found by the software PockDrug.^40^ (b) SASA distributions over configurations with selected contact patterns: 0 indicates a contact surely not formed, and 1 indicates a contact surely formed. Top: upper pocket; bottom: distal pocket.

Our analysis on the relevant contacts also unveils the presence of another interesting pocket far from the catalytic site, in the interface region between domains II and III (right-hand side of the table in Figure 2a). The five relevant contacts in this region are Arg^131^-Thr^199^, Arg^131^-Asp^289^, Pro^132^-Thr^196^, Asp^197^, and Thr^198^-Asn^238^, Tyr^239^-Leu^287^. This region, which we call the *distal pocket*, has been previously identified and screened for docking and has been predicted as a potential druggable target.^41,42^ It has also been suggested as a target for allosteric inhibition of the catalytic activity.^43,44^ Coherently, the predicted druggability score is 0.65 ± 0.08. Experimental confirmation of the viability of the distal pocket as a target comes from crystallographic fragment screening.^42^ Among the hits that were identified, three are particularly interesting. Fragment Mpro-x0390, classified as “high confidence”, is in contact with atoms from five different residues, among which four are involved in the relevant contacts mentioned above. Fragment Mpro-x0464, also classified as “high confidence”, is in contact with 11 residues, among which six are involved in the relevant contacts. Fragment Mpro-x1163, classified as “correct ligand but with weak density”, is in contact with nine residues, among which five are involved in the relevant contacts. With a completely different approach, the database Pocketome^45^ identifies for the coronavirus M^pro^ a bindable pocket in the distal region, with two possible ligands (entry R1AB_SARS2_P6); this pocket includes residues Pro^132^, Thr^196^, Thr^198^, Asn^238^, and Tyr^239^, all involved in the five relevant distal pocket contacts. Alternatively, many other algorithms have been developed for the detection and scoring of druggable pockets.^46−52^ We decided to further benchmark our findings by running the pocket detection software fpocket.^53^ While for most structures the analysis does not detect any pocket in the distal region, the structures in the core set of state m1:9 display two pockets in contact with various residues in the distal region, even if with low druggability. Finally, we analyze the whole trajectory with the software MDpocket,^54^ which quantifies in terms of a frequency grid the points involved in accessible pockets: the frequency value ranges from 0 if a point is never found along the trajectory in an open pocket to 1 if it is always found. The software assigns low values to the distal pockets: this suggests that the distal pocket is observed as a transient site, which makes its detection nontrivial. With the aim of verifying the presence of allosteric effects involving the distal pocket, we focus on the above-mentioned contacts in this region. We compute, e.g., the distribution of the PDA and of the SASA restricted to the frames in which the contact patterns are those of states m2:4 and m2:5 in the table in Figure 2a. Despite all considered residues being far from the binding pocket, the distributions of the PDA and of the SASA are sizably different in the two conditions. This suggests that if these five contacts could be forced to be formed or broken according to the desired pattern, e.g., by a drug-like compound, one could influence the PDA and the SASA, controlling indirectly the access to the reactive site. Comparing the table in Figure 2a and Figure 1b, a good candidate for allosteric drugging seems to be the contact pattern of state m1:9: (0,0,0,1,1). Interestingly, the PDA and SASA distributions obtained by selecting only the first three of the five contacts, namely (0,0,0), do not differ significantly from those with all five contacts involved (see e.g. Figure 3b).

We finally analyze the conservation of the residues involved in all the proposed contact patterns in the sequences of proteins belonging to the same family as M^pro^. We perform a multiple sequence alignment of our sequence (from PDB 6Y84(39)) with all the sequences in the Pfam^55^ seed of the corresponding family, Coronavirus endopeptidase C30 (Pfam entry PF05409). Similarly to ref (56), we find that many of the residues involved in the proposed target sites are conserved in all or most of the sequences (see Supporting Information) and furthermore all of them are conserved in the sequence of Human SARS coronavirus (SARS-CoV).

In conclusion, our data analysis approach allowed us to identify 18 putative metastable states of the M^pro^ of SARS-CoV-2. We characterized these states in terms of their structural differences, identifying some contacts which are selectively formed or broken in the different states. We believe that this analysis brings insight on the molecule’s conformational changes which might prove useful for the design of pharmaceutical inhibitors. Our analysis approach is useful especially for understanding (and eventually controlling) the global dynamics of a protein, since treats the region of the catalytic cavity and any other part of the protein within the same framework. We stress that the same kind of analysis can easily be applied to any other candidate target proteins, due to its extreme generality.

Based on this analysis we propose some possible target sites for the design of drug-like molecules, some of which directly in contact with the flaps regulating the access to the enzyme’s active site, some located in the distal pocket at the interface between domains II and III of the monomers. We provide evidence of allosteric effects connected to such pocket, and we propose as drug target simply three contacts whose inhibition is correlated to a reduction in the access to the catalytic site; a more refined drug design could yield even stronger catalytic inhibition. We show that all three proposed target sites are comprised in pockets with high druggability score according to the software PockDrug. We find that all residues involved in the proposed target sites are conserved between the M^pro^ of Human SARS-CoV and Human SARS-CoV-2 and that many of them are conserved in most sequences in the seed of the Pfam family to which they both belong. We interpret this as a comforting indication for the validity of our proposed targets. Moreover, the conservation of all such residues might suggest that mutations are unlikely, thus hopefully the displayed allosteric mechanisms are resistant to possible future mutations. A further possible interesting way to validate the viability of the predicted pockets as potential drug targets, especially of the distal pocket, would be analyzing the effect of mutations in that region on the catalytic activity. Finally, a dynamical docking simulation would be the next step to assess our findings from a more accurate biochemical standpoint.

To summarize, the added value provided by our analysis is two-fold. First, and most importantly, we provide the structure of the state which should be targeted for drug design. This structure does not coincide with the crystallographic structure, and not even with the most likely configuration observed in the MD simulation—indeed some crucial tertiary contacts which are formed in the crystal are not formed in the structure we propose, and these contacts form and break dynamically along the trajectory. Available bioinformatic tools for searching druggable cavities do not normally provide hints on the structural rearrangement which should be induced by the drug to modify the properties of the catalytic cavity, as we are instead able to do. In the second nontrivial insight provided by our analysis, it unveils high mobility in the distal pocket region, excluding the presence of relevant conformational changes coupled with the accessibility of the catalytic dyad in other sites. Even if we cannot exclude that allosteric effects may arise even from other pockets, our findings suggest prioritizing these targets among the wealth of putative binding sites found by automatic scanning.

The structures of the putative metastable states described in this work are available in Supporting Information for independent structural analysis and for targeted drug design which, we hope, will be performed by groups with the appropriate competencies.

## Supporting Information Available

Definition of PDA; mobile and relevant dihedrals analysis; detailed structural description of the metastable states; mutation and conservation analysis of relevant residues within the same protein family (PDF)
Structures of the putative metastable states described in this work (ZIP)
