# Supplementary material for: Candidate Binding Sites for Allosteric Inhibition of the SARS-CoV-2 Main Protease from the Analysis of Large-Scale Molecular Dynamics Simulations
Source: J Phys Chem Lett. 2020 Dec 11;12(1):65–72. doi: 10.1021/acs.jpclett.0c03182 (PMC7755075; doi:10.1021/acs.jpclett.0c03182)
Supplement: Supplementary file 1 — jz0c03182_si_001.pdf [file jz0c03182_si_001.pdf]

Supporting Information: Candidate Binding Sites for Allosteric Inhibition of the SARS-CoV-2 Main Protease from the Analysis of Large Scale Molecular Dynamic Simulations

Matteo Carli,[†] Giulia Sormani,[†] Alex Rodriguez,[‡] and Alessandro Laio[*,†]

†*SISSA, Via Bonomea 265, 34136 Trieste, Italy*

‡*ICTP, Str. Costiera, 11, 34151 Trieste, Italy*

E-mail: laio@sissa.it

Definition of the Pocket Doorway Area: PDA

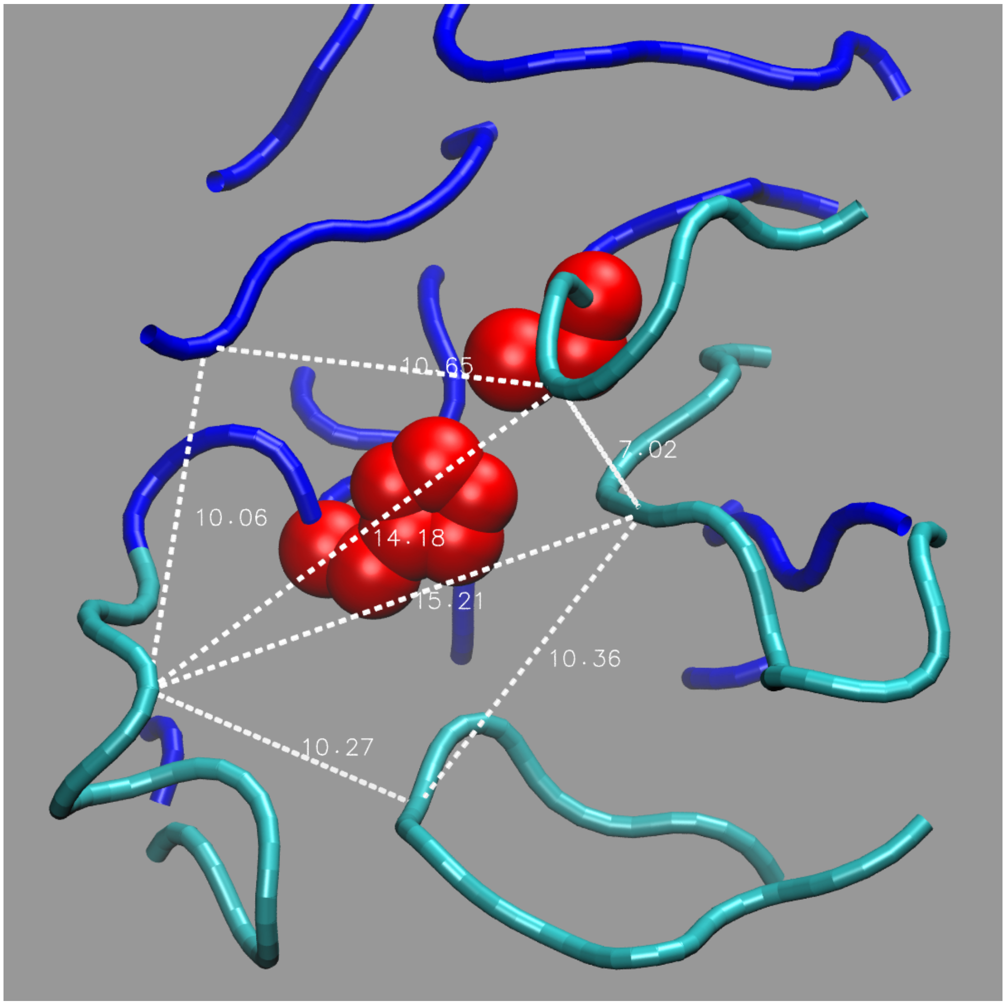

Figure S1: Visualisation of the backbone (in dark blue) of the residues surrounding the catalytic dyad (in red) and thus shaping the enzyme's binding pocket. In light blue the most flexible loop surrounding the cavity are represented: the left and upper flap, the linker and right loop. In white dashed lines, the segments connecting the five Cα atoms so to define the three triangles whose total area we call PDA. Such triangles are: Thr^{25}-Ser^{46}-Gly^{143}, Ser^{46}-Gly^{143}-Met^{165} and Gly^{143}-Met^{165}-Arg^{188}. The segment labels report distances in Å.

Mobile and relevant dihedrals

Table S1: Selected ψ backbone dihedral angles. The first three column refer to the three most flexible loops, which are the ones controlling the access to the catalytic pocket. The remaining columns refer to other isolated dihedrals, selected due to their high variability throughout the 18 states. For each row, the average over the configurations of the corresponding state is considered. For a better readability, we adopt a ternary labelling: if $-\pi/2 < \psi < pi/6$ the angle is labeled as α; if $\psi < -11/12\pi$ or $\psi > \pi/2$ as β; in all other cases the angle is labeled as c. Dihedrals whose label does not vary in any of the states of a given monomer are reported in light gray colour.

state ID	Ile^{43}-Pro^{52} loop	Phe^{140}-Cys^{145} loop	Phe^{185}-Tyr^{201} loop	2	61	153	154	168
m1:1	αββαααααcβ	βββαββ	ββcββcββββββββββα	β	β	c	α	α
m1:2	αββαααααcβ	ββcαββ	ββcβββββββββcβββα	β	β	c	α	α
m1:3	αααααcααcβ	cαβααβ	ββcβcββββββcβββα	β	β	β	c	α
m1:4	ααααβcβαβc	cβcαββ	ββcβcβcββββββββα	β	β	β	c	α
m1:5	ααααβcβαcβ	cαβααβ	ββcβcβcβββββcβββα	β	β	β	α	α
m1:6	αββαααβαcβ	cαβααβ	ββcβββcββββββββcα	c	β	β	c	α
m1:7	ααααβcββcβ	βββαββ	ββcβcβcβββββcββcα	β	β	β	α	α
m1:8	αβαcβcαβββ	βββαββ	ββcβββcββββββββββα	β	β	c	α	α
m1:9	ααααβcββcβ	ββcαββ	ββcββββββββββββα	β	β	β	α	α
m1:10	ααβαβαββcβ	ββcαββ	ββcββββββββββββα	β	β	β	α	α
m1:11	ααααβcββcβ	cβcαββ	ββcβββcββββββββββα	β	β	β	α	α
m2:1	αββαααααcβ	βββααβ	ββcβcβββββββcβββα	β	β	β	c	α
m2:2	αββαααααcβ	βββααβ	ββcβcββββββββββββα	β	β	c	α	α
m2:3	αββββααβββ	βββcαβ	ββcβcβββββββcββcα	α	α	c	α	α
m2:4	βββββαβαβα	cββααβ	ββcβββcβββββββββα	α	β	c	α	α
m2:5	αββββαβββα	ββββββ	cαβααααααβββββββα	β	β	c	α	β
m2:6	αββαβαcαcβ	βββcαβ	ββcβcβββββββcβββα	c	β	c	α	α
m2:7	αβββββαβββ	βββααβ	ββcβββcβββββcβββα	β	β	c	α	α

Structural description of the metastable states

We here present a description of all 18 metastable states in terms of their local contact structure and backbone arrangement and of the two observables SASA and PDA. Some parts of the description might be redundant with the main text of the manuscript.

From the analysis of the maximum residence time it is clear that states 1 and 2 of both m1 and m2 are among the longest-lived metastable states. All four are in fact very similar to the crystallographic structure (PDB 6Y84): they all have the left flap and the

linker loop in contact between each other (cont. Met^{49}-Gln^{189}); the left flap is closed (cont. Glu^{47}-Leu^{57} broken, cont. Thr^{25}-Cys^{44} formed) and the linker loop stretched towards it (cont. Leu^{167}-Arg^{188} broken), covering the lower part of the binding pocket. The contact and backbone structures of states m2:1 and m2:2 are almost identical and even a visual inspection with the software VMD confirms the two states can be considered in practice as the same metastable state (even the SASA and PDVA have compatible values within errorbars); the difference between states m2:1,m2:2 and m1:1,m1:2 is the fact that the latter two have the F140-C145 loop (we call it *upper flap*) tilted downwards (contacts 28 vs 143-144 and 118 vs 142 not formed, dihedral 144 in β instead of α configuration), which hides the catalytic Cys^{145}, resulting in a slightly lower SASA and PDVA. The differences between m1:1 and m1:2, instead, are mostly in the linker loop, which in m1:2 is wider in proximity of the pocket (cont. 185-186 vs 192 not formed) and narrower towards the end (contacts 132 vs 196 and 197-198 vs 238 formed, 131 vs 199 not formed).

Two other states which are similar to each other in terms of contact structure are m2:6 and m2:7. The upper flap is not bent downwards (dihedral 144 in α configuration, as most of the states in m2), leaving some SASA for the catalytic Cys^{145}. In m2:7 the left flap is more stretched towards the linker loop, and the linker loop is open wider, granting slightly lower PDVA and SASA. In both cases, however, the catalytic dyad is quite accessible.

Then there are states m1:9 and m1:10 which are very similar in their contact and backbone structure, with the exception of the left flap, which is much more open in state m1:10. States m1:9 and m1:10 (especially the former) are then both structurally similar to m1:7: the only difference among the contacts is 132 vs 196, which is formed in m1:7 and not formed in m1:9 and m1:10, allowing the lower loop to be more flexible. In all three states the upper flap is tilted downwards; surprisingly, despite the fact that the left flap is wide open, two out of these three states are detected as closed by our observables. In m1:9 the side-chains of the residues in the loops surrounding the binding pocket are oriented towards the catalytic dyad, causing such state to rank among the lowest in SASA;

moreover, cont. 285 vs 285* in this state is not completely formed (*n* configuration). State m1:7 ranks among the lowest in PDVA and as the lowest in SASA; the reason lies in the sidechains of the lower and left flaps, in particular of Thr^{45} and Gln^{189}, which form a contact and effectively close the access to the reactive site.

Another couple of similar states is that of m1:4 and m1:11: they characterised by a very open left flap (cont. 47 vs 57 formed) and the upper flap still tilted downwards. They rank among the most open in PDVA but not very high in SASA, due to the upper flap and to sidechains orientation (especially in m1:11). State m1:4 is among the only three states in which the contact of the dimer interface (cont. 285 vs 285*) is a little looser than in the others.

The remaining states do not present close similarities to others in terms of contact structure; we describe them in approximate order of decreasing openness of the catalytic pocket. The most open state according to both PDVA and SASA is m2:4; its upper flap is not tilted downwards and is retracted from the pocket, distancing from the β-sheet M162-G170 loop (we call it *right loop*), leaving cont. 138 vs 172 not formed; the left flap is very open (although the dihedrals of this loop are quite variable among the configurations of such state); the linker loop is slightly contracted and wide (cont. 131 vs 199 and 132 vs 196 not formed), not stretching towards the left flap as in other closed or partly-closed states; all of the above play to leave the catalytic dyad well exposed.

State m1:8 also ranks very high in PDVA and in SASA, despite the upper flap tilted downwards. The left flap is very open, although dihedrals 43-46 are not all in α configuration; their particular arrangement ($\alpha\beta\alpha c$), however, grants that the biggest sidechains of the left flap are not oriented towards the binding pocket. The linker loop is not strerched towards the left flap, but rather down, towards the interface with the solvent; it is quite open (dihedral 189 in c instead of β configuration) in proximity of the pocket and all its sidechains do not obstruct the access to the cavity (in particular those of Arg^{188} and Gln^{189}, responsible for a low SASA in other states).

State m1:5 is characterised by an having the left flap open (although less than e.g. state m1:4 and m1:11), with cont. 47 vs 57 formed, and the upper loop not tilted. The right loop leans slightly towards the tip of linker loop (Arg^{188}), causing cont. 138 vs 172 to be broken and cont. 167 vs 188 to be formed between the sidechain of Leu^{167} and the backbone of Arg^{188}. All other contacts far from the pocket are formed. The linker loop leans towards the left flap rather than down.

In state m1:3 the position of the upper flap and of the right loop are approximately the same as in m1:5. The linker loop stretches a bit more toward the left flap, causing contacts 132 vs 196 and 197-198 vs 238 to be broken. The left flap is closed, forming contact 49 vs 189 with the linker loop. The lower part of the pocket results closed, but the catalytic dyad is left quite exposed from above, which yields a central position in both SASA and PDVA ranks.

Also state m1:6 leaves the pocket quite accessible from the top and covered from the bottom. The linker loop is quite open, while the left flap is closed and stretched towards it. The peculiar shape of the left flap brings the α-carbons of Ser^{46} and Arg^{188} very clpse together, which results in a very low PDVA (second lowest in the ranking).

State m2:3 ranks as the third lowest in both SASA and PDVA. Cys^{145} is not well covered, but on the other hand His^{41} is less accessible than in most other states. As most m2 states, m2:3 has the upper flap flat and cont. 138 vs 172 not formed. The linker loop is not stretched, leaving the contacts with residue Arg^{131} unformed or partly unformed. The left flap is really closed and stretched towards the linker loop and its dihedrals are arranged in such a way that cont. 49 vs 189 is not formed; however, these two most mobile loops have a contact between Glu^{47} and Gln^{189}.

Finally, state m2:5 is the one with the lowest PDVA and is among the lowest-ranked in SASA. Its conformation is quite peculiar: the linker loop is all retracted and coiled (it is the only state of m2 forming cont. 167 vs 188). The left flap is all stretched towards the linker loop (cont. 49 vs 189 formed), which, with the contribution of the sidechains, almost

completely covers the catalytic His^{41}. The upper flap, rather than being flat or tilted down, is oriented upwards, causing a deformation in the II domain which allows cont. 138 vs 172 to be formed. Remarkably, m2:5 is one of the three states with cont. 285 vs 285^* not tightly formed.

Mutation and conservation of relevant residues within the same protein family

Table S2: Amino acid 1-letter code of relevant residues in the Human SARS-CoV2 $3CL^{pro}$ (from PDB 6Y84) and of the corresponding residues in the other proteins in the seed of the same Pfam family (Coronavirus endopeptidase C30, Pfam entry PF05409), obtained via multiple sequence alignment. The sequence IDs reported as column headers refer to the sequence of Human SARS-CoV2 $3CL^{pro}$, in the first non-header line of the table. Analysing the table, all relevant contacts are conserved between the M^{pro} of Human SARS-CoV2 and Human SARS-CoV. Particularly stable within the protein sequences appear to be the residues corresponding to: Tyr^{118}, Arg^{131}, Asp^{289}, Leu^{287}. Furthermore, quite recurrent are Asn^{142}, Thr^{196}, Asp^{197}.

Source Organism	47	57	118	142	131	132	196	197	198	199	238	239	287	289
Human SARS-CoV2	E	L	Y	N	R	P	T	D	T	T	N	Y	L	D
Human SARS-CoV	E	L	Y	N	R	P	T	D	T	T	N	Y	L	D
Murine coronavirus	A	L	Y	C	R	S	Q	D	Y	T	G	F	L	D
Human coronavirus 229E	T	E	Y	N	R	T	A	N	Q	M	G	F	L	D
Feline coronavirus	T	E	Y	A	R	S	T	N	V	M	S	F	L	D
Avian infectious bronchitis virus	S	V	Y	A	R	S	P	D	N	L	G	F	F	D
Thrush coronavirus HKU12	K	I	Y	N	Q	T	T	F	Q	Y	S	F	F	C
